# Immune checkpoint inhibitor-induced aseptic meningitis and encephalitis: a case-series and narrative review

**DOI:** 10.1177/20420986211004745

**Published:** 2021-03-29

**Authors:** Laure Thouvenin, Timothée Olivier, Giuseppe Banna, Alfredo Addeo, Alex Friedlaender

**Affiliations:** Oncology Department, Geneva University Hospital, Geneva, Switzerland; Oncology Department, Geneva University Hospital, Geneva, Switzerland; Oncology Department, Portsmouth Hospitals NHS Trust, Portsmouth, UK; Oncology Department, Geneva University Hospital, Geneva, Switzerland; Oncology Department, Geneva University Hospital, 4 Rue Gabrielle-Perret-Gentil, Geneva, 1205, Switzerland

**Keywords:** aseptic meningitis, autoimmune meningitis, immune checkpoint inhibitors, immune toxicity, meningoencephalitis, neurological adverse events

## Abstract

**Background:**

Along with the increasing use of immune checkpoint inhibitors comes a surge in immune-related toxicity. Here, we review the currently available data regarding neurological immune adverse events, and more specifically aseptic meningitis and encephalitis, and present treatment and diagnostic recommendations. Furthermore, we present five cases of immunotherapy-induced aseptic meningitis and encephalitis treated at our institution.

**Recent findings:**

Neurological immune-related adverse events, including aseptic meningitis and encephalitis, secondary to checkpoint inhibitors are a rare but complex and clinically relevant entity, comprising a wide range of diseases, most often presenting with symptoms with a wide range of differential diagnoses. Our case-series highlights the challenges of such entities and the importance of properly identifying and managing aseptic meningitis and encephalitis.

**Summary:**

Checkpoint inhibitor-induced meningoencephalitis warrants prompt investigations and treatment. Properly diagnosing aseptic meningitis, encephalitis, or mixed presentations may guide the treatment decision, as highlighted by our case-series. After rapid exclusion of alternative diagnoses, urgent corticosteroids are the therapeutic backbone but this could change in favour of highly specific cytokine-directed treatment options.

**Plain language summary:**

**Aseptic meningitis and encephalitis with immune checkpoint inhibitors: a single centre case-series and review of the literature**

Over the course of the past decade, checkpoint inhibitors have revolutionized cancer care. With their favourable toxicity profile and potential for durable and deep responses, they have become ubiquitous across the field of oncology. Furthermore, combination checkpoint inhibitors are also gaining ground, with increased efficacy and, unfortunately, immune-related toxicity. While there are guidelines based on extensive clinical experience for frequent adverse events, uncommon entities are less readily identified and treated. Neurological immune-related adverse events secondary to checkpoint inhibitors are a rare but complex entity, comprising a wide range of diseases, most often presenting with aspecific symptoms. In this paper, we discuss a single institution case-series of patients with autoimmune aseptic meningitis and encephalitis, and we perform a narrative literature review on this subject. We conclude with our treatment recommendations based on available evidence.

## Introduction

Over the course of the past decade, immune checkpoint inhibitors (ICIs) have revolutionized medical oncology, with hitherto unseen long-term, durable responses and improved overall survival. Modern ICIs are divided into two main categories: anti-cytotoxic T lymphocyte antigen-4 (CTLA4) antibodies (such as ipilimumab and tremelimumab) and antibodies targeting the programmed death 1 (PD-1) axis, including anti-PD-1 (pembrolizumab, nivolumab, cemiplimab) and its ligand (anti-PD-L1; durvalumab, atezolizumab, avelumab). Others are under development and may soon affect the treatment landscape.^1^

Since their initial use in melanoma, ICIs have rapidly gained ground, expanding to metastatic non-small cell lung cancer (NSCLC), urothelial cancer, and many other cancer types.^2,3^ While initially investigated as monotherapy, ICI combinations have proven their efficacy and have already become front-line options in melanoma and kidney cancer.^4,5^ The possibility to combine chemotherapy with ICIs has further expanded the indications of these drugs, ensuring that virtually all fit patients with metastatic NSCLC and head and neck squamous cell carcinoma could be exposed to front-line ICIs,^6–9^ with the exception of most patients with actionable mutations.^10–12^ Advanced or metastatic urothelial carcinoma management has also been modified, with ICIs being used as second-line therapy, and recently also in the maintenance setting after first-line chemotherapy.^13^ ICIs are also explored in the neoadjuvant setting in some tumour types^14^ (ClinicalTrials.gov identifiers: NCT03425643 and NCT03158129).

ICIs are globally considered safer than chemotherapy, because of lower rates of grade 3 or 4 adverse events. Nevertheless, they are not without risk, with potential serious immune-related adverse events (irAEs), with an estimated 2% of treatment-related mortality.^15^ Neurological immune-related adverse events (NAEs) secondary to checkpoint inhibitors are a rare but complex entity, comprising a wide range of diseases, most often presenting with nonspecific symptoms. In this paper, we analyse currently available data about aseptic meningitis and encephalitis and summarize all known cases of these adverse events, including those in our centre. We conclude with management recommendations for checkpoint inhibitor-induced aseptic meningitis and encephalitis.

## Methods

We searched PubMed with the following keywords for all available cases reported in English (“immune checkpoint inhibitor”, “ipilimumab”, “nivolumab” “pembrolizumab”, “atezolizumab”, “durvalumab”, “neurologic toxicities”, “neurologic immune-related adverse events”, “meningitis”, “encephalitis”, “ meningoencephalitis”). We also collected all cases of aseptic meningitis or/and encephalitis among patients treated with ICIs from our immunotoxicity board. We identified five cases between 2015 and 2019, described below (Table 1).

**Table 1.** Autoimmune meningoencephalitis case reports from Geneva and the literature.

Patient (Geneva^*^)	Tumour histology	ICI	NAE (symptoms)	Diagnosis workup	NAE treatment	NAE evolution	Tumoral response
**F – 46 yo (** ^*,76^ **)**	Stage IV uveal melanoma	Ipi 3 mg/kg 4C	**Meningoencephalitis gr 3** (headaches, hearing loss, morning dizziness, low fever)	LP: high cc (91% ly), high protein level, cytology and flow cytometry negative. Ab−. Brain MRI: regressing hypophysitis signs.	Methylpred i.v. 4 mg/kg/d for 6 d, then oral dexa 0.6 mg/kg/d for 2 d, then 0.5 mg/kg/d and slow tapering over 6 weeks. Ipi discontinued.	Improvement in a few days. *Relapse* 1 week after cessation of steroids: oral dexa 4 mg/d. No improvement, increased to 12 mg/d. Improvement in a few days, tapering over 3 months.	PR for 2 years. At progression pembro: no NAE; PD. Deceased.
**M – 70 yo (** ^*^ **)**	Stage IV RCC	Nivo 1 mg/kg 1C	**Meningoencephalitis gr 4** (neck pain and stiffness, fever, confusion, gait disturbance, dysphasia)	LP: high cc (66% ly), high protein level. Brain MRI: possible ventriculitis.	Dexa i.v. 0.6 mg/kg/d (10 mg 4×/d) one day, then methylpred i.v. 1.8 mg/kg/d (125 mg/d) for 7 d. Then oral prednisone 1,5 mg/kg/d, very slow tapering over 6 months. Nivo discontinued.	Improvement in a few days. But very slow steroid decreasing because of *relapsing symptoms* (neck pain, fever) several times.	PR for 7 months. At progression pazopanib.
**F – 44 yo (** ^*^ **)**	Stage IV MSI colorectal carcinoma	Ipi 1 mg/kg + nivo 3 mg/kg 3C	**Meningitis gr 3** (fever, headaches, photophobia, anorexia)	LP: high cc (92% ly), high protein level.	Methylpred i.v. 2 mg/kg/d (125 mg/d) for 3 d, then oral prednisone 1.5 mg/kg/d (100 mg/d) po. Tapering over 6 weeks. Ipi discontinued. Nivo withhold, and shortly resumed after prednisone ended.	Symptoms quickly getting better. No relapse.	Dissociated radiological response, decreasing CEA.
**M – 82 yo (** ^*^ **)**	Hodgkin’s lymphoma, second relapse	Pembro 200 mg 1C	**Meningoencephalitis gr 3** (confusion, impaired speech, fever, gait disturbance)	LP: high cc (91% ly), high protein level, flow cytometry negative. Brain MRI: multiple contrast lepto-meningeal enhancements.	Methylpred i.v. 1 mg/kg/d for 5 d, then oral prednisone 1 mg/kg/d. Tapering over 3 months. Pembro discontinued.	Symptoms getting better in a few days.	CR.
**F – 70 yo (** ^*^ **)**	Stage IV SCLC	Atezo 1200 mg 3C	**Anti-Hu limbic encephalitis gr 3** (confusion, anxiety, hallucination, disorientation, memory trouble)	Brain MRI: bilateral hippocampal T2 hyperintensities. LP: high cc (96% ly), elevated protein level. Anti-Hu Ab+. Serum: anti-Hu Ab+. EEG: encephalopathy with interictal irritant focus predominant on the right and ictal focus predominant on the left.	Methylpred i.v. 2 mg/kg/d once, then 1 gr/d for 3 d. Because of the steroid-refractory state: IVIG (0.4 g/kg/d). Rituximab i.v. 1 gr + methylpred i.v. 250 mg. Then steroids tapering over 2 weeks. At relapse, IVIG (0.4 g/kg/d) + methylpred i.v. 1 mg/kg for 5 d. Then transition to oral prednisone 1 mg/kg/d, with no tapering; IVIG once a week; rituximab/6 months. Atezo discontinued.	No clinical benefit with steroids, and then with IVIG too. Discrete improvement under rituximab and methylpred. *Relapse* 2 months after steroids end. Persistent memory trouble and dependency for daily life activities.	PR for 5 months, then PD.
M – 60 yo^34^	Stage IV pleomorphic lung carcinoma	Nivo 3 mg/kg 3C	**Anti-Hu limbic encephalitis gr 4** (memory disturbance, evolving to muscular weakness, then loss of consciousness and respiratory arrest)	Brain MRI: high intensity area in the temporal lobe, thalamus, central aqueduct and spinal cord. LP: high cc, elevated protein level. Serum: Anti-Hu Ab+ (before and after nivo).	Methylpred i.v. high dose + plasmapheresis twice. Nivo discontinued.	Improvement of the brain MRI intensities, but the patient dit not regain consciousness.	Initially PR. Died 6 months after the encephalitis from PD.
M – 68 yo^53^	Stage IV RCC	Ipi 1 mg/kg + nivo 3 mg/kg 3C; Then nivo 1 mg/kg 1C	**Meningoencephalitis gr 3** (fever, speech disturbance, confusion, drowsiness)	LP: high cc (99% mononuclear), high protein level, normal glucose level. Brain MRI: diffuse dural enhancements. *EEG*: encephalopathic changes.	Oral prednisone 100 mg/d for 7 d. Tapering over 1 month. ICIs discontinued.	Full recovery.	SD (at 9 months FU).
F – 19 yo^53^	Stage IV BRAF mutated melanoma	Ipi + nivo 3C	**Meningoencephalitis** gr UK	LP: high cc (97% mononuclear), high protein level. Brain CT: normal.	Dexa i.v. 20 mg/d (10 mg 2×/d) for 8 d. Tapering over 1 month. ICI withhold, restarted 3 months after NAE resolution.	Full recovery.	PD
F – 26 yo^53^	Hodgkin’s lymphoma, second relapse	Nivo 1C	**Anti-Ma2 limbic encephalitis** gr UK	Brain MRI: FLAIR signal changes in mesial temporal lobes. *EEG* : encephalopathic changes. Serum: Anti-Ma2 Ab+.	Dexa i.v. 20 mg/d, decreased over 12 d. Then oral prednisone 10 mg/d for 14 d, then 5 mg/d. Nivo discontinued.	Full recovery.	SD (at 2 months FU).
F – 71 yo^30^	Stage IV SCLC Received PCI.	Ipi 3 mg/kg + nivo 1 mg/kg 1C	**Anti-Hu limbic encephalitis gr 3** (short term memory deficits worsening, disorientation, dysexecutive disorders)	Brain MRI: severe abnormalities in both hippocampi with contrast-enhancing lesions. LP: high cc (87% ly), high protein level, cytology negative. Anti-Hu Ab+ (and an uncharacterized antibody against Purkinje cells).	Methylpred i.v. 1000 mg/d 5 d. At relapse, natalizumab and increase in steroids. ICIs discontinued.	*Relapse* with steroids tapering. Neurologic improvement over 2 months and steroids weaned off completely, after natalizumab.	Durable oncologic response.
F – 50 yo^35^	Stage IV melanoma	Ipi 1 mg/kg + nivo 3 mg/kg 1C	**Anti-NMDAR encephalitis gr 4** (fever, myalgia, nausea, syncopal episodes, memory loss, gait disturbance, abnormal behaviour, progressing to stuporous state)	Brain MRI: known metastasis, no change. LP: high cc (100% ly), normal glucose and protein levels. Cytology negative. IgG NMDAR Ab+. EEG: occasional seizures with left temporo-occipital origin.	Methylpred i.v. 1000 mg/d + IVIG 0.4 mg/kg/d for 5 d. Rituximab i.v. 1 g 2 doses. ICIs discontinued.	No improvement initially. After rituximab, slow improvement over 4 weeks, to a full recovery.	PR for 12 months.
M – 60 yo^35^	Stage IV SCLC	Ipi 3 mg/kg + nivo 1 mg/kg 1C	**Anti-glial nuclear limbic encephalitis gr** 4 (memory loss, gait disturbance evolving over 2 weeks to: disorientation, right arm dysesthesia, lethargy)	Brain MRI: new T2 hyperintensity in the right mesial temporal lobe. Serum: hyponatremia, anti-glial nuclear Ab+. LP: high cc(85% ly), high protein level, normal glucose level.	Oral prednisone 60 mg/d. ICIs discontinued.	Fast improvement of the neurologic symptoms.	PR for 5 months. Deceased after PD.
M – 53 yo^36^	Non-Hodgkin’s B-cell lymphoma, 1st recurrence	Nivo (40–80 mg biweekly) 2 months (maintenance after ASCT)	**Encephalitis gr 3** (diplopia, dysarthria and gait ataxia initially following nivolumab infusions, and then being permanent)	Brain MRI: small, scattered, T2 and FLAIR hyperintense, contrast-enhancing lesions dorsal to the left lateral ventricle and in the midbrain and brain stem. LP: lymphocytosis, high protein level. Flow cytometry negative. Ab− in serum and CSF. Supraventricular stereotaxic biopsy: inflammation, but no sign of lymphoma, vasculitis or viral (JC, EBV) infection.	Methylpred i.v. 1000 mg/d for 5 d, 1 course of IVIG, two courses of IV cyclophosphamide 750 mg. Then oral dexa 8–12 mg/d. Nivo discontinued.	Slow improvement, with persistent fluctuating dysarthria and ataxia. *Relapse* of symptoms with dexamethasone weaning.	CR for 18 months after ASCT, then relapsed and chose to commit assisted suicide.
F – 70 yo^37^	Stage IV RCC	Ipi 1 mg/kg + nivo 3 mg/kg 2C	**Meningitis gr 2** (headaches, nausea, dizziness)	Brain MRI: normal. LP: high cc (99% ly), high protein level, normal glucose level. Cytology negative.	Prednisolone i.v. 1 mg/kg/d. Tapering over 1 month. ICIs withheld. Restarted when 10 mg/d oral prednisolone. ICI discontinued after the other AEs.	Fast improvement of symptoms. After the 3^rd^ cycle: *adrenal insufficiency* treated with hydrocortisone. Then, *relapse* of a *meningitis gr2*, with *hepatitis*, treated with prednisolone i.v. 1 mg/kg/d with efficacy.	CR after the 3^rd^ cycle.
M – 56 yo^38^	Stage IV uveal melanoma	Ipi 3 mg/kg 4C	**Meningoencephalitis gr 3** (malaise, nausea, fatigue, fever, upper respiratory tract symptoms, evolving to gait imbalance, decreased mental status, myoclonic jerking of the limbs, auditive and tactile hallucinations)	Brain MRI: normal; and then diffuse dural thickening enhancement. LP: high cc (96% ly), high protein level, normal glucose level. EEG: marked encephalopathy. Dural biopsy: minimal perivascular inflammation, acute and chronic.	Methylpred i.v. 160 mg/d. Tapering over 4 weeks. Ipi discontinued.	Dramatic improvement in 48h.	Unknown.
M – 55 yo^39^	Stage IV lung adenocarcinoma, with previously treated brain metastases	Pembro 200 mg 11C	**Meningitis gr 3** (violent headaches, photophobia)	Brain CT: normal. LP: increased opening pressure, high cc (30% ly), high protein level, normal glucose level. Brain MRI: normal.	Dexa i.v. 10 mg, then 24 mg/d (6 mg 4×/d). Transition to oral steroids and then tapering. Pembro discontinued after 11C for the hepatitis.	Headaches disappeared in 24h.	CR.
F – 53 yo^57^	Stage IV melanoma	Ipi 3 mg/kg + nivo 1 mg/kg 2C	**Meningoencephalitis gr 3** (fever, aphasia, dizziness, fatigue, instability to walk, slurred speech)	Brain MRI: normal. EEG: diffuse marked cerebral slowing. LP: high cc (86% ly), high protein level, normal glucose level.	Dexa i.v. 40 mg/d (10 mg/6 h). ICIs discontinued.	Improvement at day 3.	PD. After a PD under dabrafenib-trametinib: Pembro with no NAE.
M – 61 yo^57^	Stage IV melanoma	Ipi 3 mg/kg + nivo 1 mg/kg 4C; Then nivo 3 mg/kg 1C	**Meningoencephalitis gr 4** (slow progression of altered mental status to unresponsive state)	Brain CT: normal. Brain MRI: aspecific. EEG: seizure activity. LP: high cc, high protein level.	Methylpred i.v. 125 mg 2×/d, and antiepileptic drugs. Methylpred i.v. 1 g/d + IVIG 1 g/kg/d. Then transition to oral prednisone 1 mg/kg/d. ICIs discontinued.	Refractory seizure. After increased steroids and IVIG, improvement to full recovery.	PD in 4 months.
M – 57 yo^57^	Stage IV melanoma	Nivo 3 mg/kg 6C; Then ipi 3 mg/kg 4C; Then nivo 3 mg/kg 8C	**Meningoencephalitis gr 3** (headaches, then confusion and altered mental status)	Brain CT and MRI: normal. EEG: no seizure but diffuse slowing and biphasic waves. LP: high cc with lymphocytosis.	Methylpred IV. Then tapering. ICIs discontinued.	Resolution on 6 days.	Near CR.
F – 83 yo^57^	Stage IV melanoma	Nivo 3 mg/kg 4C	**Meningoencephalitis gr 5** (change in mental status and suspected seizure; 10 days later inferior limbs weakness, evolving to comatous state)	Brain MRI: residual melanoma in the nasolacrimal duct. Some hypersignal in the supratentorial white matter. LP: normal cc, high protein level, and normal glucose level.	No other steroid treatment than her maintenance therapy (oral prednisone 20–0–10 mg).	Deceased in 5 days.	
F – 58 yo^57^	Stage IV melanoma, with prior brain radiotherapy	Ipi 4C: PD. Nivo 3 mg/kg 2C	**Encephalitis gr 3** (fatigue, disorientation, incoherence, aphasia)	Brain MRI: stable metastatic disease. LP: high cc (92% ly), Ab−.	Methylpred i.v. 100 mg/d for 6 d. Increasing to dexa i.v. 200 mg/d + IVIG 0.4 g/kg/d for 5 d. Then tapering. Nivo discontinued.	No improvement. After dexa and IVIG slow improvement over 11 days.	PD. Deceased (3 months afther the NAE).
UK^74^	Stage IV melanoma	Ipi 2C	**Meningitis gr 3** (headaches, drowsiness, nausea, vomiting)	Brain MRI: UK. LP: few lymphocytes.	No steroids. Ipi discontinued.	Spontaneous and complete improvement in 10 days.	SD. PD at 6 months. Deceased (46 months after the NAE).
UK^74^	Stage IV melanoma	Ipi 2C	**Meningoencephalitis gr 3** (delirium)	LP: normal Brain MRI: UK	Oral prednisolone. Ipi discontinued.	Complete resolution in 8 weeks.	PD. Deceased (14 months after the NAE).
UK^74^	Stage IV melanoma	Ipi + nivo 2C	**Meningitis gr 2** (headaches and nausea)	LP: reactive lymphocytes. Brain MRI: UK.	No steroids. ICIs withhold, then restarted.	Complete resolution at 7 weeks.	PR for 16 months, then PD.
F – 64 yo^40^	Stage IV clear cell ovarian cancer, relapsed	Nivo 8C	**Encephalitis gr 4** (fever, delirium, stiff arms and legs)	Brain MRI: normal. LP: normal. Serum: severe hypocalcaemia, GAD65 Ab+ (low specificity for autoimmune encephalopathy).	Methylpred i.v. 1200 mg/d (3 g 4×/d) + 10 sessions plasmapheresis.	Slow improvement. Several months before return to baseline.	
F – 71 yo^41^	Stage IV lung adenocarcinoma, relapsed	Pembro 6C	**Anti-Rib meningoencephalitis gr 3** (diplopia, unsteady gait, urinary incontinence, tremors, lower limb paraesthesia)	Brain MRI: normal Spine CT: normal LP: high cc (lymphocytes), high protein level, anti-Ri Ab+.	Dexa tapering over 12 weeks. At first relapse, i.v. steroids + rituximab. Then gradual prednisone tapering over months. At second recurrence, cyclophosphamide being considered. Pembro discontinued.	Complete resolution at 8 weeks. *Relapse* 3 weeks after ending steroids (recurrent diplopia). *Recurrent relapse* (diplopia) under prednisone at 4 months.	CR.
M – 20 yo^42^	Hodgkin’s lymphoma, refractory	Nivo 3 mg/kg 3C	**Meningo-cerebellitis gr 3** (headaches, diplopia, confusion, nausea, vomiting, ataxia, dysmetria)	Brain CT: cerebellar oedema. Brain MRI: diffusely oedematous cerebellum with patchy enhancement, signs of early tonsillar herniation. LP: unsuccessful; at day 6: high cc (94% ly), high protein level, normal glucose level, flow cytometry normal.	Dexa 32 mg/d (8 mg/6 h), tapering over 4 weeks. Nivo discontinued.	At day 6, no more nausea. Full recovery, except for mild diplopia.	PR.
F – 60 yo^25^	Stage IV melanoma, prior brain surgery and radiotherapy	Ipi 3 mg/kg 4C (PD 3 months after the last dose) Pembro 2 mg/kg 1C	**Brain stem encephalitis gr 5** (deceased)	Autopsia: Diffuse and nodular microglial activation in the brain particularly in the brainstem with lymphocytic infiltrates CD8^+^ (absence of tumoral or infectious aetiologies).		Deceased unexpectedly 2 weeks after pembro.	
F – 67 yo^26^	Stage IV squamous NSCLC	Nivo 3 mg/kg 2C	**Encephalitis gr 5** (disorientation, aphasia, intermittent somnolence)	EEG: no seizure, but diffuse slowing. LP: high cc (lymphocytes), high protein level, normal glucose level. Brain MRI: unremarkable. Brain MRI at deterioration: multiple and confluent cortical and subcortical FLAIR hyperintensities. LP at deterioration: high cc, high protein level, neuronal Ab-. Serum: neuronal Ab-.	IVIG 0.5 g/kg for 5 d. At deterioration, antiepileptic drugs + methylpred i.v. 1 g/d for 2 d.	Initially slight improvement. But in a few days developed stupor with focal seizures. No improvement with increased therapy. Palliative care.	Deceased.
M – 63 yo^43^	Stage IV RCC	Nivo 300 mg/2 w 6C	**Anti-PNMA2 meningoencephalitis gr 5**(change in behaviour, uncontrolled choreatic movements)	Brain MRI: symmetrical, pathologically increased signal within the basal ganglia. LP: mild inflammatory change, anti-PNMA2 Ab+, flow cytometry−. Autopsia: focal lymphocytic meningitis of the entire brain and cervical spinal cord.	Methylpred i.v. 2 mg/kg/d. At deterioration, antipsychotic drugs + Infliximab 5 mg/kg/d. At the new deterioration, antibiotic, and antipsychotic and corticosteroids reintroduce.	Further deterioration in the choreatic movements, and development of a paranoid hallucinatory syndrome. No clinical benefit of the increased therapy, but discharged (patient decision). 3weeks after: fever and GCS 5; despite therapy introduction: clinical deterioration with pneumonia and low GCS, leading to death.	PR. Deceased.
F – 66 yo^44^	Stage IV lung adenocarcinoma	Nivo 4 months	**Encephalitis gr 5** (hemiballismus evolving to bilateral ballismus, dysarthria, orobucco-lingual dyskinesia)	Brain MRI: symmetric T2 hyperintense and T1 hypointense basal ganglia abnormalities. LP: normal cc and glucose level, mildly elevated protein level, cytology−. One unclassified paraneoplastic Ab+.	Methylpred i.v. 1 g/d + plasmapheresis, both for 5 d. As refractory, haloperidol and olanzapine. Then IVIG 2.5 g/kg/d + prednisone + rituximab 1000 mg once + tetrabenazine (20 mg 3×/d).	Refractory, and continue to decline despite increased therapy. Palliative care.	Deceased.
F – 44 yo^44^	Stage IV lung adenocarcinoma	Nivo 3 mg/kg 5C	**Anti-GAD65 limbic encephalitis gr 4** (progressive altered mental status, nausea, vomiting, partial seizure).	EEG: left temporal slowing, interictal discharges. Brain MRI: T2 signal hyperintensities of the bilateral mesial temporal lobes. LP: high cc (97% ly), normal protein and glucose levels, cytology−, anti-GAD65 Ab+ (in the serum too).	Methylpred i.v. 1 g/d for 5 d, followed by 5 days of plasmapheresis + antiepileptic drugs. Rituximab 1 gr IV. Rituximab maintenance (1 gr/6 m) Nivo discontinued.	Initially improved seizure. But then, deterioration (refractory seizures, developing ataxia, vertigo and gait disturbance), improved with rituximab. Residual vertigo and gait ataxia; brain MRI: normalized.	UK.
M – 78 yo^45^	Stage IV squamous NSCLC	Nivo 3 mg/kg 14C	**Encephalitis gr 4** (apathy, aphasia, progressing on 24 h to GCS 7, recurrent extremities myoclonuses)	Brain CT: stable intracranial epidermoid tumour of the left temporal lobe. EEG: moderate background slowing and focal delta slowing over the left temporal region with singular sharp waves in this region. Brain MRI: unremarkable. LP: high cc (lymphocytes), high protein and lactate levels, slightly reduced glucose level. Serum: Ab−.	Antiepileptic drugs. At day 12, methylpred i.v. 1.33 mg/kg/d. Tapering over 9 weeks. Nivo discontinued.	Fluctuating GCS (4–13) for 11 days. With steroid introduction, improvement in 24 h to a GCS 15. Return to baseline neurologic status.	PR for 9 months. Deceased at 9 months of bacterial pneumonia.
M – 51 yo^46^	Stage IV squamous NSCLC, prior brain surgery and radiotherapy	Pembro 8 months	**Meningoencephalitis gr 4** (fever, headaches, unsteadiness in walking, 1 week later: dizziness, difficulty in communication, GCS 10, neck stiffness and Kernig sign +)	Brain MRI: no change. LP: high cc (lymphocytes), high protein level. EEG: slow waves in the right frontal robe. Serum: Ab−.	Prednisolone 2 mg/kg/d. Tapering by 10% per week. Pembro discontinued.	Fast improvement in the level of consciousness, but unsteady for walk during 2 weeks.	SD for at least 1 year.
M – 69 yo^46^	Stage IV uveal melanoma	Pembro 4C	**Encephalitis gr 3** (fever, confusion, weakness)	LP: high cc (30% ly), high protein level, normal glucose level.	Methylpred i.v. 2 mg/kg/d. Transition to oral prednisone, with tapering over 5 weeks. Pembro discontinued.	Improvement of the mental status rapidly after a single dose of steroid. Fever and testicular pain resolved with steroids.	SD for 6 months.
F – 51 yo^47^	Stage IV melanoma	Pembro 10 mg/kg/2 w 36C (maximum according by the study)	**Encephalopathy gr 3** (1 month after the end of the ICI: muscular pains progressing over 6 weeks with then headaches, floaters in visual field. 6 weeks later: tethered waxy skin, puffiness of the face, muscular pain, confusion, incontinence, gait disturbance)	Serum: high eosinophil count. Arm MRI: marked fascia oedema of the musculature. Brain MRI: hyperintense white matter foci subcortical, ovoid lesions perpendicular to the ventricles and involving the corpus callosum (DD: ischaemic or demyelination process). LP: normal.	Methylpred i.v. 2 mg/kg/d, after 10 d increased to 1 g/d because of the brain MRI results. After 10 days, transition to oral prednisone 60 mg/d, and taper of 5 mg/week.	Brain MRI after 10 d of steroids: increased enhancement in the previous lesions: more suggestive of multiple infarctions (treated with aspirine 81 mg/d). After 20 d, resolution of the confusion and fasciitis; gait, weakness and deficits in proprioception improved more slowly.	CR.
M – 66 yo^48^	Stage IV melanoma	Dacar-bazine + ipi 4C (PD) Lambro 2 mg/kg/3 w 6C	**Encephalitis gr 2** (after 4C: ataxia, vertigo, left arm numbness; after 6C: left arm twitching = partial seizure)	Brain MRI: normal at 4C; at 6 C: FLAIR hyperintensities bilaterally in the claustrum, right frontal and left occipital lobes. LP: mild pleiocytosis, high protein level, cytology−. EEG: periodic epileptic form discharges. Brain biopsy: diffuse microglial activation, focal perivascular inflammation with lymphocytic infiltrates (aspecific CNS inflammation), no malignancy.	Antiepileptic drugs. Lambro discontinued, then anticonvulsant tapering.	No recurring seizures. Full recovery. Brain MRI at 2 and 4 months: resolution of the FLAIR changes.	PR.
M – 74 yo^49^	Stage IV NSCLC, prior brain radiotherapy	Nivo 1C	**Encephalitis gr 4** (progressing altered mental status, dysarthria, weakness of lower limbs, urinary retention)	Brain CT: no acute changes. EEG: mild slowing, no seizure activity. LP: inconspicuous, normal glucose level, Ab-.	Dexa IV. At relapse, methylpred i.v. for 5 d. Tapering over 6 weeks. Nivo discontinued.	Initial improvement, but steroids discontinued for agitation. *Relapse* of confusionnal state and mental status waxed and wane. After 3 d of methylpred return to baseline.	UK.
F – 54 yo^50^	Stage IV lung adenocarcinoma, with brain metastases	Nivo 3 mg/kg 2C	**Cerebellitis gr 3** (dizziness, nausea, nystagmus, cerebellar ataxia)	LP: high cc (100% ly), high protein level. Ab−. Cytology−. Serum: Ab−. Brain MRI: no abnormalities (brain metastasis disappeared after EGFR therapy).	Dexa 10 mg/d for 3 d. Methylpred 1 g/d for 3 d. Transition to oral prednisolone starting at 30 mg/d and gradually tapering. Nivo discontinued.	Initial worsening symptoms. Improvement under methylpred. Ataxia and nystagmus almost completely resolved.	Deceased 2 months later from pneumonia.
M – 64 yo^51^	Stage IV melanoma	Pembro 52 weeks	**Limbic encephalitis gr 3** (progressive memory decline since therapy initiation)	Brain CT: no metastasis. Brain MRI: symmetrical T2 hyperintensities, with atrophy, in hippocampi, anterior temporal lobe, and insula. LP: high cc (lymphocytes), high protein level. Ab−. Cytology−. EEG: no seizure activity.	Initially pembro withheld for 1 month, then restarted. Methylpred i.v., followed by oral prednisolone tapering. Pembro discontinued.	Persistent significant cognition decline. Despite methylpred stable cognition deficit. Serial LP: lowering of the inflammatory process. Brain MRI: no changes.	Deceased from his oncological disease.
M – 56 yo^78^	Melanoma, inguinal lymph node metastasis	Adjuvant ipi 10 mg/kg 4C	**Meningo-radicolo-neuritis gr 4** (dizziness, cervicalgia, headaches, progressing over 13 d to falls, dysarthria, dysesthesia, then progressing to severe gait ataxia)	Serum: high eosinophil level. LP: high cc (99% ly), high protein level, low glucose level. Ab−. Brain MRI: normal. Spine MRI: global enhancement of nerve roots (arachnoiditis).	Oral prednisone 80 mg/d. Methylpred i.v. 1 g/d for 3 d. Then transition to IVIG 0.4 g/kg/d for 5 d + oral prednisone 1 mg/kg/d for 4 months. Ipi discontinued.	Worsening neurologic symptoms to tetraplegia. With methylpred slow improvement over 1 month, but still unable to walk. Almost complete recovery after 24 months.	UK.
M – 64 yo^79^	Stage IV prostate adenocarcinoma	Ipi 10 mg/kg/21 dfor 4C, then/3 months for 3C	**Encephalitis gr 3** (3 months evolution of adynamia, memory disturbances, disorientation, hallucination, focal seizures)	Brain CT: normal. EEG: generalized slowing with slow theta and delta waves. LP: cc and glucose level normal, high protein level. Ab−. Serum: Ab−. Brain MRI: mild microangiopathic changes, normal pituitary gland.	Antiepileptic drug stopped seizures, but other symptoms persists. Methylpred i.v. 1 g/d for 3 d. Transition to oral prednisolone 100 mg/d, and tapered after one week to 60 mg/d, then tapered over 4 weeks by 10 mg/week. Ipi discontinued.	At day 3, began to improve. Almost full recovery, except for subtle memory deficits.	Radiologic and biologic CR for at least 2 years.
F – 39 yo^80^	Stage IIIA melanoma	Adjuvant ipi 10 mg/kg 3C	**Meningo-encephalo-myelitis gr 3** (initially headaches, flu like symptoms)	LP: elevated opening pressure, high cc (lymphocytes), Ab−, cytology−. Brain and spine MRI: leptomeningeal enhancement and pituitary enlargement. At relapse: Brain and spine MRI: recurrent leptomeningeal and cranial nerve enhancement and expansion of the cervical cord and T2 hyperintense cord signal alteration with abnormal patchy intramedullary enhancement. LP: lymphocytosis, high protein level.	Methylpred i.v. 1 mg/kg/d, then transition to oral tapering prednisone over 8 weeks, until hydrocortisone. ICI discontinued. Relapse treated by IVIG 1 g/kg/d + methylpred i.v. 1 mg/kg/d for 5 d. Then infliximab i.v. 5 mg/kg + prednisone 1 mg/kg/d. Followed by 2 more infliximab doses/15 d, and oral prednisone 60 mg/d for 2 months, then tapered gradually by 10 mg/15 d over 3 months.	Rapid improvement. *Relapse* at 3 months (lower limb weakness, incontinence), scant improvement with IVIG and methylpred. But improvement with infliximab and prednisone. Near complete neurologic recovery. *Brain MRI*: decreased leptomeningeal enhancement.	UK.
M – 57 yo^81^	Stage IV SCLC	Ipi + nivo 5C	**Acute cerebellitis gr 3** (diplopia, dysarthria, nystagmus, ataxia)	Brain MRI: marked cerebellar oedema, diffuse high intensity in the cerebellar cortex. LP: high cc (lymphocytes), high protein level, normal glucose level, cytology−.	Methylpred i.v. 1 g/d for 3 d. Then six cycles of plasmapheresis added. ICIs discontinued. At 10 and 14 weeks, two cycles of i.v. rituximab 375 mg/m^2^.	Slight improvement. *Brain MRI*: edema improvement. *LP* : inflammation reducing. But severe cerebellar symptoms persisting, with limited improvement under rituximab.	SD.
M – 51 yo^82^	Stage IV melanoma, with brain metastases	Ipi 3 mg/kg 1C	**Meningitis gr 2** (severe headaches, fever)	LP: increased opening pressure, high cc and protein levels, cytology−. Brain MRI: known cerebellar metastasis.	Oral dexa 8 mg/d. UK if ICI continued.	Complete recovery in a few days.	SD for 10 months.
M – 44 yo^83^	Stage IV RCC	Nivo 240 mg 1C	**Encephalitis gr 4** (deteriorated mental status, hallucinations, aggressiveness, fever)	Brain MRI: unremarkable.	Nivo discontinued. Same dexa therapy continue (for bone pain).	Improvement in a week.	Deceased from metastatic RCC.
M – 78 yo^84^	Malignant pleural mesothelioma	Nivo 1C	**Anti-Ma2 induced encephalitis gr 3** (At day 9 fever, anorexia; at day 22 somnolence; at day 41 nystagmus, ophthalmoplegia)	Brain MRI: normal. LP: high cc and protein levels, cytology−. Serum: Anti-Ma2 Ab+ (pre-existing). Brain MRI at day 41: T2 hyperintensity mesencephalon and medial thalami.	Nivo withheld, then discontinued. Steroids when deteriorating.	Major improvement with steroids.	PR.
F – 28 yo^85^	Hodgkin’s lymphoma	Nivo escalating doses (80 mg on day 1, 120 mg (2 mg/kg) on day 14)	**Encephalitis gr 2** (headaches worsening since 2 weeks, nausea, dizziness)	LP: high cc (90% ly), high protein level, normal glucose level. Brain MRI: unremarkable.	Nivo withheld, not restarted as CR. Methylpred i.v. 1 mg/kg/d for 3 d, transition to oral dexa 4 mg/d for 3 d, 2 mg/d for 2 d, 1 mg/d for 2 d, then stop.	Rapid improvement, and complete recovery in 24h. After dexa cessation, *rash* (DD: nivo *versus* GVHD) treated by prednisone 2 mg/kg/d for 45 days.	CR.
F – 45 yo^86^	Stage IV melanoma, with brain metastases	Ipi 3 mg/kg 3C (+ WBRT and stereo-tactic radio-surgery)	**Meningoencephalitis gr 3** (confusion, dizziness, headaches, nausea, vomiting, dysmetria)	LP: high cc and protein levels, Ab−. Brain MRI: no changes.	Dexa 8 mg/d. Methylpred i.v. 1 mg/kg/d for 5 d at deterioration. Then IVIG 0.4 g/kg/d for 5 d. Ipi discontinued.	Small clinical improvement. With deterioration (fever, worsening neurologic symptoms) 2 days later. No improvement with methylpred. But major improvement with IVIG association.	UK.
M – 67 yo^87^	Stage IV melanoma	Pembro 2 mg/kg C10	**Anti-CASPR2 limbic encephalitis gr 3** (short term memory loss, emotional lability, confusion, altered speech)	LP: high cc (lymphocytes), anti-CASPR2 Ab+. Serum: Anti-CASPR2 Ab+. Brain MRI: T2 hyperintensity medial temporal lobes and contrast enhancement. EEG: background slowing, with intermittent delta slowing.	Antiepileptic drugs. Methylpred i.v., followed by high-dose oral prednisolone, slow tapering. Pembro already discontinued.	Improvement of cognitive function. Resolution of MRI changes.	UK.
M – 46 yo^88^	Stage IV extraskeletal myxoid chondro-sarcoma	Cemi 3 mg/kg/14 d 5C	**Anti-Hu limbic encephalitis gr 4** (increased anxiety and depression, memory loss)	Brain MRI: prominent abnormalities in the left medial temporal lobe. LP: high cc (83% ly), normal protein and glucose levels, cytology−. Anti-Hu Ab+.	Methylpred i.v. 1 g/d for 5 d + IVIG 1 g/kg/d for 2 d. Transition to oral prednisone 60 mg/d, with 10 mg/d tapering. At relapse, methylpred i.v. 1 gr/d for 6 d, followed by oral prednisone 60 mg/d and rituximab i.v. 375 mg/m^2^.	Stable, no more mental status deterioration. MRI improvement. *Relapse* at 7 weeks (rapid confusion, dysarthria, weakness). No improvement under prednisone and rituximab. Became obnunted and developed pneumonia. Palliative care.	SD. Deceased from pneumoniae.
M – 70 yo^89^	Stage IV lung adenocarcinoma	Nivo 3 mg/kg 4 months	**Encephalitis gr 3** (impaired gustatory sense, anorexia, action tremor, difficulty walking, fever)	Brain MRI: nonspecific T2 hyperintense lesions in the cerebral white matter. EEG: diffuse slow waves, no seizure. LP: high cc and protein level, cytology−.	Methylpred i.v. 500 mg/d for 3 d. Repeated 4 weeks after, transition to slow tapering regimen of oral prednisolone. Nivo discontinued.	Improvement in walk and tremor only after the methylpred therapy at 4 weeks.	PR, stable for more than 1 year.
M – 75 yo^90^	Stage IV SCLC, prior brain surgery and radiotherapy	Ipi 3 mg/kg + nivo 1 mg/kg/21 d 4C	**Encephalitis gr 3** (convulsions leading to altered metal status)	Brain MRI: no new changes. LP: high cc and protein level, cytology−. Ab−. EEG: no seizure	Methylpred i.v. 500 mg/d (10 mg/kg/d) + IVIG 0.4 g/kg/d. Adding rituximab i.v. 375 mg/m^2^. ICIs discontinued.	Initially no improvement. Several days after rituximab, neurologic improvement. Stable after 1 month.	PD.

Ab, antibody; AE, adverse event; ASCT, autologous stem cell transplantation; atezo, atezolizumab; C, cycle; cc, cell count; cemi, cemiplimab; chemo, chemotherapy; CNS, central nervous system; CR, complete response; CT, computed tomography; d, day; DD, differential diagnosis; dexa, dexamethasone; EBV, Epstein Barr virus; EEG, electroencephalogram; EGFR, Epidermal Growth Factor Receptor; F, female; FLAIR, Fluid-attenuated inversion recovery; FU, follow up; GCS, Glasgow coma scale; gr, grade; GVHD, graft *versus* host disease; h, hour; HCB, haematocephalic barrier; HRT, hormone replacement therapy; ICI, immune checkpoint inhibitor; Ipi, ipilimumab; irAE, immune-related adverse event; i.v., intravenous; IVIG, i.v. immunoglobulin; JC, John Cunningham virus; lambro, lambrolizumab; LP, lumbar puncture; ly, lymphocytes; M, male; methylpred, methylprednisolone; MRI, magnetic resonance imaging; MSI, microsatellite instability; NAE, neurological adverse event; nivo, nivolumab; NSCLC, non-small cell lung cancer; PCI, prophylactic brain irradiation; PD, progressive disease; PR, partial response; pembro, pembrolizumab; RCC, renal cell carcinoma; SCLC, small-cell lung cancer; SD, stable disease; St, stage; UK, unknown; w, week; WBRT, whole-brain radiotherapy; yo, year old.

For the narrative literature review, we used the sources found on PubMed with the above search, as well as data from international guidelines.

## Cases

First case: a 46-year-old woman with metastatic (liver and peritoneum) uveal melanoma started ipilimumab at 3 mg/kg. After two cycles, she developed headaches with episodes of fainting. Blood tests found inaugural central hypothyroidism, which quickly led to panhypopituitarism. This immune-related grade 2 hypophysitis was treated with methylprednisolone and hormone replacement therapy.

After effective hormonal replacement and a switch to hydrocortisone, the ICI treatment was resumed. After the fourth cycle, the patient developed headaches with bilateral hearing loss, nausea and grade 3 asthenia. She was subfebrile and described morning dizziness. At admission, she had static cerebellar syndrome.

A brain magnetic resonance image (MRI) showed regressive sequelae of hypophysitis, without secondary lesions or bleeding.

A lumbar puncture (LP) demonstrated pleiocytosis with 91% of lymphocytes, elevated protein level, and normal cytology and flow cytometry. After the LP, antibiotics (ceftriaxone) and antivirals (acyclovir) were started with pulse intravenous methylprednisolone.

The Gram stain and viral polymerase chain reaction (PCR) tests were negative and corresponding treatments were stopped. Onco-neuronal antibodies were not detected. The diagnosis of grade 3 aseptic meningoencephalitis was retained.

After 48 h of corticosteroids, the patient was afebrile, and hearing loss and dizziness began to improve. A second LP reported a decreasing pleiocytosis and normal protein levels.

Given the positive clinical course occurring 7 days after intravenous (i.v.) steroids, they were replaced with oral prednisone and slowly tapered. After 5 weeks, despite ongoing prednisone, she presented recurring headaches, bilateral hearing loss and ataxia, with fever.

No brain MRI or LP was repeated, but 4 days before the symptoms reappeared, a LP showed a rise in central spinal fluid (CSF) lymphocytes and protein levels. Corticosteroids were increased and antibiotic and antiviral treatments resumed, with no improvement after 4 days. Bacterial cultures and viral PCRs were negative, allowing to a further increase in steroid dose finally leading to a slow positive evolution. Infliximab was considered but not given, and steroids were maintained for 4 weeks then tapered over 4 weeks.

Ipilimumab was permanently discontinued, and the patient had a partial response for 2 years. Upon progression, she was treated with pembrolizumab (2 mg/kg every 3 weeks) without any neurological adverse events. ICIs were stopped at disease progression. The patient died 8 months after the initiation of this last line of treatment.

Second case: a 70-year-old man was diagnosed with metastatic (lung) renal clear cell carcinoma. He had a left radical nephrectomy and splenectomy, and begun treatment with a combination of nivolumab 1 mg/kg and ipilimumab 3 mg/kg within a clinical trial. At 5 days after his first cycle, he developed increasing neck pain, followed by fever, and finally confusion, gait disturbance and aphasia.

At day 10, he was admitted with fever, confused speech, neck stiffness and gait disturbance. An LP showed pleiocytosis with elevated lymphocytes and high protein levels. The brain scan was normal and the interpretation of the brain MRI was limited because of the patient’s agitation, but suggested ventriculitis.

Empiric treatment with acyclovir, amoxicillin and ceftriaxone was stopped when the Gram stain, culture and viral PCR results came back negative. Dexamethasone allowed for a rapidly favourable clinical course: he became afebrile, and his gait and speech improved. At 8 days after admission, a regimen of oral prednisone was initiated and the patient went home.

Steroid tapering was complicated by several recurrences of neck pain, nausea and fever of unknown origin. Prednisone was maintained at 15 mg daily for two more months and weaned over 7 months for this grade 4 immune-related meningoencephalitis. Due to the grade of the complication and according to the trial rules, no ICIs were reintroduced.

It is interesting to note that after only one cycle, the cancer was controlled for 7 months, at which point a second-line treatment with pazopanib was introduced, with an ongoing good partial response at last follow-up.

Third case: a 44-year-old woman with relapsed metastatic (muscle, skin and liver) colorectal adenocarcinoma with microsatellite instability initiated ipilimumab 1 mg/kg and nivolumab 3 mg/kg. After three cycles, she developed fever, followed by headaches, photophobia and anorexia. On admission the clinical exam was normal, except for fever. LP demonstrated a pleiocytosis with lymphocytosis, and elevated protein levels.

Acyclovir and amoxicillin were given until CSF analyses came back negative for infection, while methylprednisolone, initiated concurrently with anti-infectious treatments, was continued. Oral prednisone was started on day 3, after the fever and symptoms improved. It was slowly weaned over 6 weeks. At this point, in an asymptomatic patient who had rapidly responded to therapy, while there was a risk of relapse of the grade 3 aseptic meningitis, the potential treatment benefit outweighed the risk. Nivolumab was reintroduced, with a good response. The patient is still on treatment and has not experienced a recurrence or any new NAE.

Fourth case: an 82-year-old man with an history of stage IIA early favourable Hodgkin’s lymphoma, initially treated with two lines of chemotherapy, presented a second recurrence 4 years after the initial diagnosis. Given his good clinical condition and lack of important comorbidities, he was started on pembrolizumab at a 200 mg flat dose. At 10 days after the first dose, his wife noted progressively increasing confusion, impaired speech, and gait disturbance. On admission, he had fever, a Glasgow coma score (GCS) of 14/15, with confusion and unstable gait. The brain scan was normal for his age. LP revealed lymphocytic pleiocytosis with elevated protein level. Antibiotics (ceftriaxone and amoxicillin) and antivirals (acyclovir) were administered until CSF analyses came back negative. The concurrent methylprednisolone was continued.

A few days after the LP, a brain MRI showed multiple leptomeningeal contrast enhancements, compatible with aseptic meningitis. At 1 day after the initiation of steroids, the patient became afebrile and gradually regained normal gait and cognition in the following days. Steroids were weaned over the course of 3 months.

Because of this grade 3 immune-related meningoencephalitis, ICIs were not reintroduced. Moreover, 2 months after the single cycle of treatment, the patient had a complete radiological and metabolic response. Since then, the patient has remained asymptomatic and has been under surveillance.

Fifth case: a 70-year-old female smoker with stage II chronic obstructive pulmonary disease was diagnosed with a neuroendocrine small cell lung cancer (SCLC) with metastatic cervical nodes. Because of a poor performance status and an intercurrent infection, her first chemotherapy cycle consisted of carboplatin AUC5 alone, allowing for an improvement in her performance status. The next three cycles consisted of carboplatin AUC5 and etoposide, combined with atezolizumab 1200 mg.

At 2 days after the fourth cycle, she was admitted to the emergency room for disorientation and visual hallucinations, with an otherwise normal neurological exam. The brain MRI was compatible with limbic encephalitis, with a bilateral abnormal signalling in the hippocampus. The LP found pleiocytosis and high protein levels, and was negative for infection, so antimicrobial treatments were stopped. An electroencephalogram (EEG) showed encephalopathy with an epileptic focus on the left side, leading to the introduction of lacosamide, with rapid EEG improvement.

From day three after therapy, she received high-dose corticosteroids, but remained disoriented. Anti-Hu antibodies were found in the CSF, leading to a differential diagnosis of paraneoplastic encephalitis *versus* autoimmune encephalitis on atezolizumab. Natalizumab (anti-integrin) was considered, but rituximab was initiated a week later, leading to improvement in her memory and a second cycle of rituximab 2 weeks later. In parallel, steroids were slowly tapered until 10 mg/d.

At 2 months later, she presented with confusion. The brain MRI showed likely recurrence of limbic encephalitis, and the thoraco-abdominal computed tomography (CT)-scan showed disease stability. Corticosteroids were raised to 1 mg/kg/d, and rituximab was given every 6 months, while i.v. immunoglobulins were administered every 4 weeks. The memory problems did not resolve and upon progression 4 months later, she received palliative care and died 4 months later.

## Discussion

### Immune-related adverse event pathogenesis

ICIs cause an overstimulation of the immune system, increasing the risk of immune-mediated toxicity. While the precise physiopathology remains unknown, there are multiple hypotheses about the underlying mechanism for developing irAEs. One of them, the “hidden autoimmunity theory”, relies on the previous existence of subclinical autoantibodies, even at a low level or “hidden” by immune-tolerance. ICIs may disrupt this equilibrium, triggering an autoimmune reaction.^52,53^ While there is an estimated 27–42% risk of exacerbating a pre-existing autoimmune disease with ICIs, there are currently no predictive markers for the incidence or severity of the reaction.^23^ Moreover, given the inherently greater risk of irAEs in these groups, patients with autoimmune disease or history of transplantation (solid organ or stem cells) are excluded from most prospective ICI trials, limiting available data to retrospective series and case reports. For instance, in a series of 22 patients with kidney transplant, half experienced rejection when challenged with ICIs.^54^ Likewise, among patients previously treated with allogenic hematopoietic stem cells transplantation, ICIs increase the risks of graft *versus* host disease and mortality.^55^ These are very selected populations but highlight that ICIs should not be viewed as innocuous. Benefits and potential complications must be discussed and evaluated with each patient.

In daily practice, there may also be a higher incidence of irAEs in high-tumour-mutational-burden diseases, such as NSCLC and melanoma, due to the greater number of neoantigens in these cancer types, leading to cross-reactivity with self-antigens, also known as the “molecular mimicry theory”.^53,56,57^ The site of cancer can also lead to an increased cross-reactivity with other organs in which antigens are more similar to those in the tumour sites.^58^

The physiopathology of irAEs can vary depending on the affected organ. For NAEs, the main explanation relies on onconeural peptides that are released during cancer cell death. They trigger the formation of onconeural autoantibodies, potentially leading to organ damage.^59^ Classical paraneoplastic syndromes (PNSs), such as Ma2-associated syndromes, have drastically increased with the use of ICIs.^60^ Similarly, it is unknown whether the presence of onconeural autoantibodies, without any accompanying PNS, as can be detected in around 16% of patients with SCLC, increases the risk of developing NAEs with ICI therapies.^59^ Investigations are ongoing to evaluate whether underlying germline factors or bacterial flora may affect the host immunity and the response and toxicity to ICIs.^55^ In preclinical studies, interleukin (IL)-1, one of the main cytokines in the acute phase of inflammation, has been shown to be central in the pathogenesis of autoimmune encephalitis, by enhancing the differentiation of IL-17-producing T lymphocytes.^61^ In checkpoint inhibitor-induced aseptic meningitis, contrarily to encephalitis, onconeural antibodies have not been shown to play a role. Interestingly, brain radiotherapy does not increase the risk of NAEs.^62^

While there is no shortage of hypotheses, we are currently unable to predict who will develop irAEs, which organ will be targeted and when. Except for autoimmune disorders and transplantation, there are no identified risk factors for the development of irAEs. While it is by no means ready for clinical use in this context, artificial intelligence could provide answers. A radiomic model was described to have a strong ability to predict immunotherapy-induced pneumonitis: radiomic and pathomic biomarkers could predict irAEs in the future.^63,64^

The exact relationship between irAEs and response to therapy is uncertain. Some irAEs seem to correlate with a radiological response to ICIs, such as the association between developing vitiligo and responding to ICIs in melanoma, or skin toxicity and response as well as survival in NSCLC.^65–67^ Regarding NAEs, there are very limited data, based on small and uncontrolled observations, suggesting an increase in objective response rate (ORR) in patients who developed NAEs (ORR 50–70%) compared with those who did not (20–30%).^23^

The relationship between the use of corticosteroids and outcomes in patients receiving ICIs is not fully understood but the available data seem to demonstrate that immunosuppressive therapies for irAEs do not attenuate their efficacy, but rather that steroid use at treatment initiation is a negative prognostic factor reflecting a poor prognosis subgroup.^55,68^

### The incidence of irAEs and neurological irAEs

In a recent meta-analysis comprising 125 clinical trials of single-agent PD-1 or PD-L1 inhibitors, involving 20,128 patients, irAEs were reported in 66% of patients, with 14% grade 3 or higher.^24^ With anti-CTLA4 antibodies, irAEs develop in 60% to 85% of patients, with 10% to 29% of grade 3 or higher.^2,69^ The combination of anti-CTLA4 (ipilimumab) and anti-PD-1 (nivolumab) antibodies increases the risk of irAEs, with adverse events reported in 95% of patients, with 55% of grade 3 or higher.^69^

Every organ can be affected, but cutaneous (pruritus in 10.61% and vitiligo in 3.26% of patients), gastrointestinal (diarrhoea in 9.47% of patients) and endocrine (hypothyroidism in 6.07% of patients) irAEs are most frequent.^24^

NAEs are uncommon, developing in roughly 2–6% of patients receiving single-agent ICIs. However, with combined PD-1 and CTLA4 inhibition, this rises to 12%.^70,71^ For PD-1 inhibition alone, an early onset of symptoms a few days after treatment is common, but NAEs can also develop after several weeks of treatment.^72^

As NAEs can present with a wide range of symptoms, some of which are easily attributed to the cancer itself (fatigue, weakness, headache, dizziness), they can be difficult to diagnose and are likely underestimated. As with other irAEs, NAEs most often present as grade 1–2 toxicity, comprising mainly headaches (55%), dysgeusia (13%) and dizziness (10%). The incidence of serious (⩾grade 3) NAEs is below 1%, and this encompasses a highly heterogeneous spectrum of neurological symptoms.^57,71,73,74^ Even if any-grade NAEs are more frequent with PD-1/PD-L1 antibodies (6.1%) than with anti-CTLA4 (3.8%), anti-CTLA4 seem to confer a higher risk for serious (grade 3–4) NAEs (0.7%) than anti-PD-1/PD-L1 antibodies (0.4%).^71^ Aseptic meningitis or encephalitis develops in 0.1–0.2% of patients on ICIs.^23^

While irAEs mostly develop between 3 and 6 months after treatment initiation, NAEs arise slightly more rapidly, with a median time to NAE of three cycles (6–9 weeks) of treatment.^2^ In a Japanese ICI safety trial, aseptic meningitis developed shortly after ICI initiation (21–32 days),^75^ and some cases were even described within the first weeks of treatment.^23^

Interestingly, non-neurological irAEs could precede NAEs, and a careful history is also mandatory when suspecting an NAE.^76^

### Aseptic meningitis and encephalitis

The clinical manifestations of NAEs are broad, can be atypical, and encompass overlapping syndromes. Peripheral involvement appears to be more common than central, with peripheral neuropathy representing up to two-thirds of all NAEs, possibly linked to previously administered neurotoxic chemotherapy.^52^ Other neurological conditions reported are myopathy/myositis, myasthenic syndrome, encephalopathy/encephalitis, aseptic meningitis, myelitis, Guillain–Barre-like syndrome, as well as immune-related PNSs.

It is important to correctly evaluate the patient for any neurological symptoms and deficits before initiating ICI therapy. Subsequently, any new neurological disorder during or after ICIs should be suspected to be a NAE, and a comprehensive differential diagnosis should investigated and ruled out. In these rare and potentially serious situations, investigations and therapies must rely on an experienced multidisciplinary team.

In aseptic meningitis, the clinical presentation can mimic an infectious meningitis with headache, neck stiffness, vomiting, photophobia and fever. However, it is likely that only one or two of these symptoms are present. Encephalitis can also be aspecific, but it differs from aseptic meningitis in that it includes altered cognition or abnormal cerebral function.^77^

One key aspect is that there can be overlapping presentation. It is important to distinguish, whenever possible, meningitis from encephalitis, as their pathogenesis and management are not strictly identical. However, they usually share the same initial workup and are sometimes grouped together under the term of meningoencephalitis.^16^

Currently, as we note in our case-series and literature review, almost all cases of encephalitis have concurrent findings of meningitis. The term meningoencephalitis can be viewed as encephalitis, as opposed to pure aseptic meningitis, in which there is no sign of encephalitis (such as confusion).

The workup must rule out an infectious or metabolic cause. First, taking a medical history, evaluating current medication, and a clinical exam are critical. Then a blood test should explore any metabolic abnormalities or signs of infection.

A brain MRI is used to rule out vascular events and (progressing) metastases. Meningeal enhancement appears in half the cases of aseptic meningitis, though its absence does not preclude the diagnosis. Parenchymal enhancement can also be found in encephalitis. Non-ICI-related autoimmune encephalitis may have a normal MRI in up to 60% of cases, so imagery should not rule out this diagnosis.^17^

An LP can detect an elevated opening pressure and must include cell count, chemistry, cytology, viral PCR (including *Herpes simplex*, *Varicella zoster*, enterovirus, Cytomegalovirus, Parechovirus), bacterial direct exam (Gram staining) and cultures, and flow cytometry in the case of a haematological malignancy. With aseptic meningitis, CSF typically reveals lymphocytic pleiocytosis, elevated protein levels, and normal glucose levels. The viral and bacterial screening is negative.

Positive onconeural antibodies help to diagnose neurologic PNSs; however, their presence is not always associated with PNSs,^18,19^ and when antibodies are detected in light of neurological symptoms in patients on ICIs, we do not know whether we are facing a classical neurological PNS or an immune-related NAE mimicking one. The “hidden autoimmunity theory”, cited above, would suggest the symptoms are NAEs, as they are not compatible with the usual presentation of PNSs, with symptoms developing before the underlying cancer detection and subacute onset.^59^

Detailed examples of initial workup results can be seen in our report of all cases reported in the literature, including the five cases identified in our institution from 2015 to 2019 (Table 1).

The initial workup will have some specificity depending on the clinical presentation. For suspected aseptic meningitis, the brain MRI and the blood examination can rule out hypophysitis and explore adrenal function (MRI with a pituitary protocol, and blood cortisol and adrenocorticotropic hormone (ACTH)). For suspected encephalitis, an EEG is necessary to detect subclinical seizures.^20^

Even if all of the above-cited investigations are normal, aseptic meningitis and autoimmune encephalitis cannot formally be ruled out, thus remaining a challenging diagnosis.

### The management of aseptic meningitis and encephalitis

Management guidelines for NAE, including aseptic meningitis and encephalitis, exist.^20,21^ These guidelines are based on expert consensus, institutional experience, and review of literature and are continuously being updated.

All irAE therapy recommendations are based on case reports, expert consensus and very rare and small prospective trials. By analogy with other irAEs, it is assumed that early recognition and aggressive treatment of aseptic meningitis and encephalitis could provide a rapid response and decrease possible residual symptoms or complications.

Some physicians believe that any NAE should be considered as grade 3 or 4, given its potential to induce severe neurological impairment.^59^ However, most guidelines suggest different management protocols according to the grade of the toxicity.^20,21^

In grade 1 NAEs, ICIs could be continued with close monitoring. In grade ⩾2 NAE, ICIs should be withheld. Corticosteroids should be introduced rapidly, the dose and route of administration depending of the NAE severity: for grade 2, oral prednisolone (0.5–1 mg/kg/d), for grade 3–4, i.v. methylprednisolone (1–2 mg/kg/d).^15,20,22,57^ It is important to note that hours to days might be necessary before definitive results of viral PCR and bacterial exams are available, so concurrent antivirals and antibiotics should be administered until infection has been ruled out.

There is usually a clinical response within the first 24 h, with no or very few sequelae. Nonetheless, the mortality rate is 5–11%^52^ for encephalitis.^25,26^

For encephalitis only, in case of severe or progressing symptoms in spite of up to 2 mg/kg steroids, pulse corticosteroids (i.v. methylprednisolone 1 g/d for 3–5 days), sometimes associated with i.v. immunoglobulins (0.4–2 g/kg/d) might be necessary. If there is no improvement in the first 48–72 h under corticosteroids, this “steroid-refractory” situation may require other immunosuppressive drugs, such as rituximab or infliximab, and plasmapheresis may be considered.^20,27,28^

After a favourable clinical course, steroids are generally maintained at a high dose for 2 weeks, then slowly tapered over at least 4–6 weeks, because of the long half-life of ICIs. If the symptoms relapse, high-dose corticosteroids are reintroduced. If corticosteroid weaning is impossible, steroid-sparing therapies such as rituximab, infliximab, or cyclophosphamide, may be added.^27^

**Figure 1.**
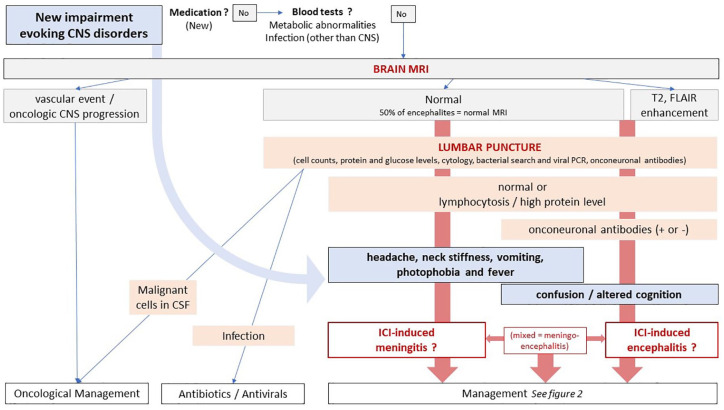
Workup of suspected checkpoint inhibitor-induced meningitis and encephalitis. CNS, central nervous system; CSF, cerebrospinal fluid; ICI, immune checkpoint inhibitor.

**Figure 2.**
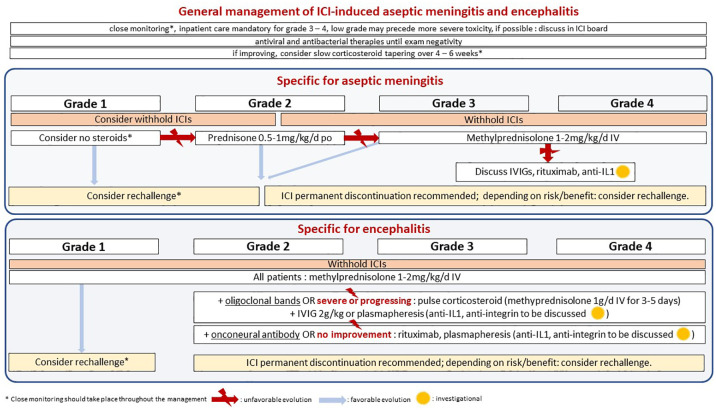
Management of checkpoint inhibitor-induced meningitis and encephalitis. ICI, immune checkpoint inhibitor; i.v., intravenous, IVIGs, i.v. immunoglobulins; po, *per os*.

American and European medical societies have management guidelines for NAEs.^20–22^ Based on those, and the published outcomes, including the present case-series, we suggest recommendations for the treatment of aseptic meningitis and autoimmune encephalitis (Figures 1 and 2) (Table 1).

Our case-series and literature review emphasize the difficulty, to differentiate between aseptic meningitis and encephalitis. This is a major issue in daily practice. Almost all cases of encephalitis presented with signs of concurrent meningitis in their cerebrospinal fluid analysis. Whether an underlying different pathogenesis and presentation should lead to a different management is not clear, and current recommendations are mostly derived from other causes of encephalitis, including paraneoplastic encephalitis. However, data are currently lacking to deviate from such guidelines.

Recently, Martins and colleagues published specific treatment algorithms for irAEs relying on their pathogenesis and on the predominant immune infiltrate in the targeted organ, which has to be biopsied.^58^ The authors evoke the possibility to treat severe irAEs with targeted cytokine-directed monoclonal antibodies or anti-integrins from the onset of symptoms, rather than using high-dose corticosteroid therapy, or to use this approach in case of poor response to steroids. For example, in aseptic meningitis and encephalitis, they suggest anti-IL-1 blockade (e.g. anakinra or canakinumab), which they suspect could be more effective than i.v. immunoglobulins or anti-CD20 antibodies (rituximab, ofatumumab, obinutuzumab, ocrelizumab). In meningitis, a neutrophilic infiltrate causes acute inflammation, partially mediated by IL-1, explaining the rationale for targeting this protein, and we have seen that IL-1 may have a central role in the pathogenesis of encephalitis.

Other strategies, such as targeting integrin 4, could be effective for meningitis and encephalitis without impairing ICI efficacy, specifically targeting lymphocyte adhesion in the blood–brain barrier.^29^ A patient with NSCLC and severe anti-Hu positive encephalitis due to ICIs was successfully treated with natalizumab, an anti-integrin 4 monoclonal antibody, without impairing the oncological outcome.^30^

This area of investigation could bring steroid-sparing treatments to the front-line therapy in patients who develop irAEs, with a short course of specific antibodies chosen according to the immune-infiltrate characteristics of the injured organ, obtained either by biopsy or even blood biomarkers.

In spite of their exciting biological rationale, these strategies need further clinical validation and are currently not recommended as upfront therapy, though they are discussed by immunotoxicity boards in the refractory setting.

### ICI rechallenge after an irAE

After any severe irAE, including an NAE, discontinuation of ICIs is recommended, particularly for grade 3–4 NAEs. There are, however, neither clear evidence nor recommendations concerning ICI rechallenge. Retrospective data have shown that among patients with grade 2 or greater irAEs, half of patients rechallenged develop irAEs, and there is generally no increase in the severity of the event.^31^ Nonetheless, the centre did not rechallenge patients with NAEs, explaining this by the fact that the consequences could be fatal.^32^

Some could advocate for more caution after encephalitis than meningitis, given its potential neurological disability. However, given the lack of strong evidence or consensus, decisions should be tailored to each patient, based on the potential risks and possible benefits.^33^ Among our cases, the two patients with encephalitis who were rechallenged with ICIs after grade 3 toxicity did not present recurring or new adverse events (Table 1). For this reason, we advocate for a tailored case-by-case evaluation, without restrictive rules about rechallenge decisions (Figure 2).

## Conclusion

With the major therapeutic advance and ubiquitous use of ICIs comes a new class of adverse events. Of these, neurological irAEs, and more precisely aseptic meningitis, encephalitis and other central NAEs are very uncommon but may be underestimated because of nonspecific symptoms and diagnostic challenges. It is important to be aware of these entities to include them in the very first differential diagnoses and initiate appropriate treatments rapidly. Current management of NAEs is mainly based on expert opinions, with initial ICI discontinuation and high-dose steroids, with differences between aseptic meningitis and encephalitis, underlying the importance of distinguishing these two entities during initial workup, when possible. Emerging therapeutic strategies based on the underlying supposed pathogenesis (cytokines, integrins) could lead to more precise and steroid-sparing therapies and need to be studied. With these new strategies under investigation, a multidisciplinary approach, with the integration of clinical and paraclinical findings, can help in the treatment decision-making process, as highlighted within our case-series.
